# Development of Equivalent Material Properties of Microbump for Simulating Chip Stacking Packaging

**DOI:** 10.3390/ma8085121

**Published:** 2015-08-07

**Authors:** Chang-Chun Lee, Tzai-Liang Tzeng, Pei-Chen Huang

**Affiliations:** Department of Mechanical Engineering, Research Center for Microsystem Engineering, Chung Yuan Christian University, 200 Chung Pei Road, Chung Li District, Taoyuan City 32023, Taiwan; E-Mails: d917716@alumni.nthu.edu.tw (T.-L.T.); mars420225@gmail.com (P.-C.H.)

**Keywords:** 3D-IC packaging, microbump interconnect, finite element analysis, equivalent material properties, analysis of variance

## Abstract

A three-dimensional integrated circuit (3D-IC) structure with a significant scale mismatch causes difficulty in analytic model construction. This paper proposes a simulation technique to introduce an equivalent material composed of microbumps and their surrounding wafer level underfill (WLUF). The mechanical properties of this equivalent material, including Young’s modulus (E), Poisson’s ratio, shear modulus, and coefficient of thermal expansion (CTE), are directly obtained by applying either a tensile load or a constant displacement, and by increasing the temperature during simulations, respectively. Analytic results indicate that at least eight microbumps at the outermost region of the chip stacking structure need to be considered as an accurate stress/strain contour in the concerned region. In addition, a factorial experimental design with analysis of variance is proposed to optimize chip stacking structure reliability with four factors: chip thickness, substrate thickness, CTE, and E-value. Analytic results show that the most significant factor is CTE of WLUF. This factor affects microbump reliability and structural warpage under a temperature cycling load and high-temperature bonding process. WLUF with low CTE and high E-value are recommended to enhance the assembly reliability of the 3D-IC architecture.

## 1. Introduction

Chip stacking assembly in three-dimensional integrated circuits (3D-ICs) packaging architecture, assisted by through-silicon via (TSV) and microbumps, has become a mainstream approach. The requirements for this process, which resulted from the development of the Internet of Things, have attracted attention. 3D-IC technology is a promising solution to integrate heterogeneous functions with high interrelated density [1]. Critical concerns about reliability issues of microbumps and 3D-IC packaging technology had been discussed [2,3]. A key reliability concern is serious warpage induced by thin TSV interposer assembly stacked chips through microbump interconnects when thermal stress/strain arises because of coefficient of thermal extension (CTE) mismatch. Accordingly, inverse analysis of warpage for stacked layers that undergo exertion of thermal cycling load is proposed [4,5]. A simple packaging structure should be able to handle difficulties in a complicated framework, such as 3D-IC packages. Thus, simulation methodologies based on finite element analysis (FEA) are essential to ensure predicted accuracy. The thermal-mechanical reliability of 3D-ICs from the viewpoint of FEA simulation is further investigated through analysis of variance (ANOVA) [6]. Combined with the validation of shadow moiré measurement [7,8], the estimated accuracy of FEA can be assured. However, FEA modeling is difficult because of the complexity of 3D-IC assemblies. Consequently, the concept of equivalent material embedded in FEA is generated. Generally, the effective material properties can be obtained in accordance with the rule of mixture; other approaches can achieve the same targets. A proper formula for effective moduli of flip chip solder is developed to predict out-of-plane deformation after packaging assemblies [9]. The aforementioned analytical model can be used to find the equivalent properties of a Si-based interposer with high-density Cu-filled TSVs [10,11].

**Figure 1:**
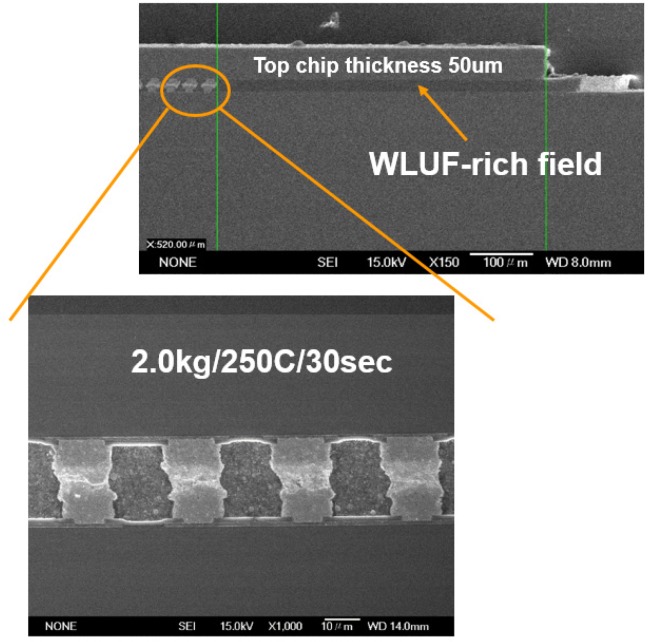
Cross-sectional view of microbump interconnects with WULF assembled through thermal compression.

The effect of environmental temperature on the warpage is not included. Few studies focus on this important factor, whereas the equivalent material property in FEA is taken into account. This paper investigates and extracts the temperature-dependent stress-strain curves and equivalent mechanical properties for the microbump array of chip stacking assemblies. To accomplish this goal, this paper considers a testing vehicle that has fine-pitch microbumps and multi-thin stacked chips; this testing vehicle is assembled by using the thermo-compressive method combined with wafer level underfill [12] (WLUF; refer to Figure 1). Dissimilar to the traditional 2.5D/3D ICs packaging structures, it should be noted that the metal-filled TSV arrays do not need to be taken into account because the major purpose of the present vehicle developed by ourselves is to meet the requirements of demonstrating the material compositions, electrical characteristics, and reliability of micro-joints after packaging assembly.

## 2. Numerical Approach of Equivalent Mechanical Properties

Scale mismatch between microbump interconnection and other structural components results in converged capability. Orthotropic constitutive behavior of the equivalent microbump unit is set in the FEA model. It should be noted that the obtainments of equivalent mechanical properties by using 3D FEA is more realistic to reflect the nature of packaging constructions. Nevertheless, a 2D system is utilized in this investigation to demonstrate the feasibility of the present approach. Based on the assumption of plane strain, the equivalent mechanical properties, namely, Young’s modulus *E_x_*, *E_y_*, Poisson’s ratio υ*_x_*, υ*_y_*, shear modulus *G_xy_*, *G_yx_*, and the coefficient of extension (CTE) α*_x_*, α*_y_*, of a microbump unit need to be calculated. The detailed explanations are as follows.

### 2.1. Equivalent Young’s Modulus of Microbump Unit

The equivalent Young’s modulus of a microbump unit is the major material property if simplification is needed for the WLUF layer with a microbump array located far from the critical corner of a real interconnected structure within FEA. The method uses weight percentage from each concerned component material; this approach could achieve the foregoing target. However, a malfunction occurs when one material of the components, for which an equivalent mechanical property is to be obtained, is either non-linear or temperature-dependent. The simulated methodology based on finite element method is considered a promising solution to this issue. Similar to the common tensile and shear test of a unique material, the applied load during the extracted process of equivalent material properties in FEA is given to emulate the practice measured situation. These equivalent material properties, including *E_x_*, *E_y_*, υ*_x_*, υ*_y_*, can be calculated in accordance with the following formula:

(1) $$\varepsilon_{x}=\frac{\Delta W}{W},\text{​ }\sigma_{x}=E_{x}\varepsilon_{x},\;\nu_{x}=-\frac{\varepsilon_{y}}{\varepsilon_{x}}$$

(2) $$\varepsilon_{y}=\frac{\Delta H}{H},\text{​ }\sigma_{y}=E_{y}\varepsilon_{y},\;\nu_{y}=-\frac{\varepsilon_{x}}{\varepsilon_{y}}$$

**Figure 2:**
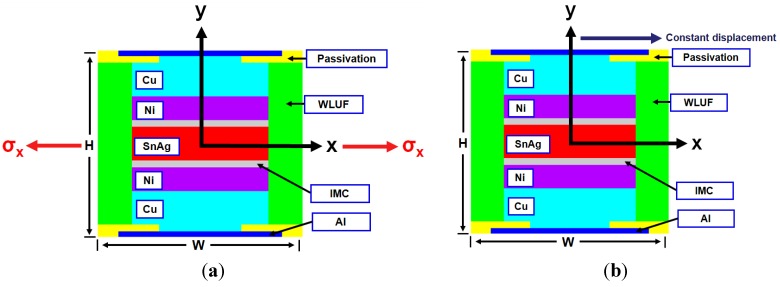
FEA model of microbump unit for the extraction of equivalent material properties: (**a**) Young’s modulus; (**b**) shear modulus.

For the boundary conditions of acquiring equivalent Young’s modulus from FEA, microbump units at *x*-directional edges are fixed on their horizontal displacements of nodes, as shown in Figure 2a. The nodal displacements at the vertical edges of the microbump unit are joined to ensure identical edge deformations of nodal displacement. To protect the FEA model from rigid body motion, all degrees of freedom at the structural center of microbump unit must be immovable. Tensile stress is loaded at the horizontal edges of the finite element model to obtain the equivalent Young’s modulus along the *x*-direction. Shrinkage is expected to occur on the vertical edges of the microbump unit. The equivalent Young’s modulus along the *x*-direction (*E_x_*) can be extracted in accordance with the ratio relationship of normal stress σ divided by the corresponding induced strain, which resulted from the incremental *x*-directional displacement divided by the characteristic dimension *W*. Similarly, to obtain the equivalent Young’s modulus along the y-direction (*E_y_*), the vertical displacements of nodes of microbump units at *y*-directional edges are fixed. Then, tensile stress is applied to the vertical edges of the microbump unit. This tensile stress is obtained as follows: the normal stress along the *y*-direction is divided by the induced *y*-directional strain, which is calculated from the y-directional displacement divided by the characteristic dimension *H*.

**Figure 3:**
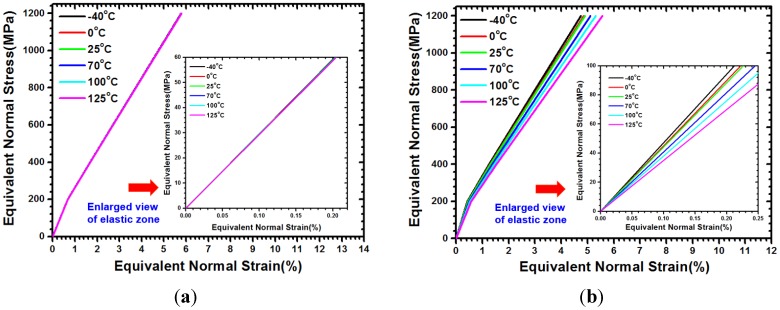
Temperature-dependent normal stress-strain curves of in-plane equivalent material for microbump unit with WLUF: (**a**) *E_x_*; (**b**) *E_y_*.

The aforementioned approach can obtain the nonlinear stress-strain curves of a microbump unit at arbitrary temperatures, as shown in Figure 3. Using the offset method with 0.2% strain, the temperature-dependent equivalent Young’s modulus along the *x*-direction is separately calculated, as listed in Table 1. Figure 3 shows that the bent curves for the plastic regions are not obvious because of the minimal given SnAg solder within the entire microbump unit. The obtained equivalent Young’s modulus along the *y*-direction of the analytic model is implemented. Results are listed in Table 2.

**Table 1:** Equivalent elastic mechanical properties of a microbump unit along the horizontal direction.

Temperature (°C)	Young’s Modulus (GPa)	Poisson’s ratio (ν*_x_*)
−40	29.6	0.37
0	29.5	0.37
25	29.5	0.37
70	29.4	0.37
100	29.3	0.37
125	29.3	0.37

**Table 2:** Equivalent elastic mechanical properties of a microbump unit along the vertical direction.

Temperature (°C)	Young’s Modulus (GPa)	Poisson’s ratio (ν*_y_*)
−40	47.1	0.3
0	44.9	0.3
25	44.1	0.3
70	40.8	0.3
100	38.0	0.3
125	34.9	0.3

The equivalent Poisson’s ratio of the microbump unit can be acquired from the displacement ratio of arbitrary bi-axes. For example, the Poisson’s ratio, which is labeled as ν*_x_*, can be obtained from the *y*-directional displacement divided by the *x*-directional displacement increment. The equivalent ν*_x_* and ν*_y_*, which are temperature-dependent, are listed in Table 1 and Table 2, respectively.

### 2.2. Equivalent Shear Modulus of Microbump Unit

The orthotropic material characteristics are generated from the mechanic viewpoint because equivalent properties of the microbump unit are introduced into the analytic model. Thus, the equivalent shear modulus for a microbump unit needs to be obtained. Based on the finite element model shown in Figure 2b, the boundary conditions of extracting the relationship between shearing stress and strain, and shear modulus *G_xy_* and *G_yx_*, are explained as follows:

(3) $$\gamma_{xy}=\frac{U_{x}}{H},\text{​ }\tau_{xy}=\frac{F_{r}}{W},\;G_{xy}=\frac{\tau_{xy}}{\gamma_{xy}}$$

(4) $$\gamma_{yx}=\frac{U_{y}}{W},\text{​ }\tau_{yx}=\frac{F_{r}}{H},\;G_{yx}=\frac{\tau_{yx}}{\gamma_{yx}}$$

**Figure 4:**
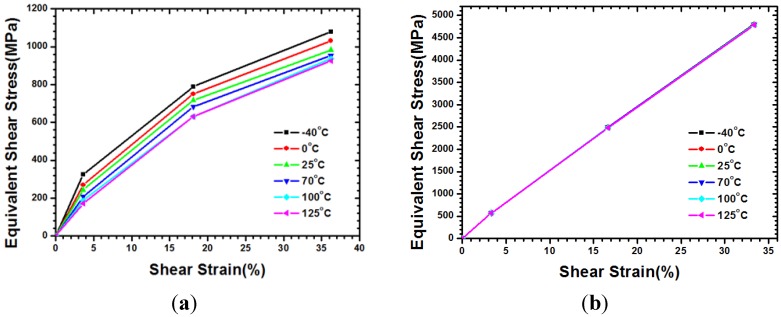
Temperature-dependent shear stress-strain curves and extraction of shear modulus of in-plane equivalent material for microbump unit with WLUF: (**a**) *G_xy_*; (**b**) *G_yx_*.

The bottom surface of the microbump unit is fixed on the ground to extract the shear modulus *G_xy_*. Identical vertical displacements of nodes on the top surface of the model are joined to ensure that they can move along the vertical direction. Then, an external constant displacement parallel to the *x*-axis is applied to the top surface. Under the assumption of a small deformation, the shear strain γ*_xy_* is determined based on the ratio of given displacement/microbump height *H*. The reacting force for each node at the bottom edge of microbump model is induced while the foregoing constant displacement is exerted; that is to say, the reacting force that divides the bottom area per thickness *W* can finally be equal to the resultant shear stress. Thus, the temperature-dependent curves of shear stress, as shown in Figure 4, can be extracted by increasing the applied shear displacement. Using a similar approach, the following explanations will help in acquiring shear modulus *G_yx_*: First, a microbump unit model with one fixed vertical edge is given. Next, a given constant displacement parallel to *y*-axis is exerted on the other edge surface. The shear strain γ*_yx_* can be obtained by the constant displacement divided by the microbump characteristic width *W*. Similarly, to obtain the Young’s modulus from normal stress-strain curves, curves that are smaller than shear strain 0.2% are considered linearly elastic. Subsequently, the shear modulus can be acquired in accordance with Hooke’s law. Table 3 lists both the *G_xy_*, and *G_yx_* at several temperature levels within a range between −55 and 125 °C.

**Table 3:** Equivalent shear modulus of a microbump unit along the horizontal direction.

Temperature (°C)	Shear Modulus (GPa) (*G_xy_*)	Shear Modulus (GPa) (*G_yx_*)
−40	8.9	14.9
0	7.5	14.9
25	6.7	14.9
70	5.7	14.9
100	5.2	14.9
125	4.7	14.9

### 2.3. Equivalent CTE of Microbump Unit

The mechanical property of CTE is one of the major concerns in applications of packaging materials. Equivalent CTE is a significant property under a change in temperature and can be used to describe the mechanical characteristics of the temperature dependence of the microbump unit. The equivalent CTE for a microbump unit can be obtained by using the following formula and explanations.

(5) $$\varepsilon_{x}=\frac{\Delta W}{W},\text{​ }\alpha_{x}=\frac{\varepsilon_{x}}{\Delta T}$$

(6) $$\varepsilon_{y}=\frac{\Delta H}{H},\text{​ }\alpha_{y}=\frac{\varepsilon_{y}}{\Delta T}$$

**Figure 5:**
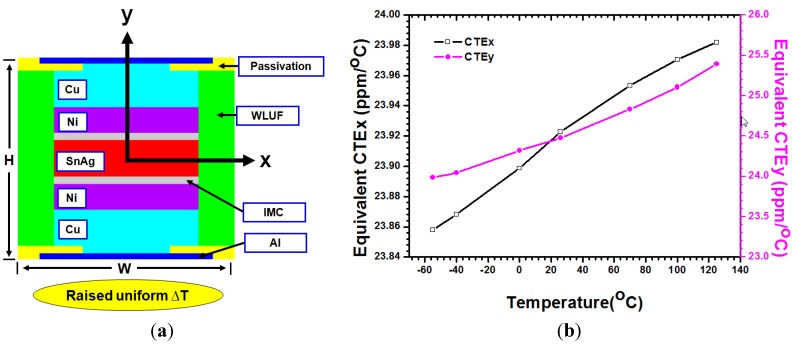
Graphical explanation and temperature-dependent curves for equivalent CTE of a microbump unit with WLUF: (**a**) FEA model of extracting equivalent CTE; (**b**) temperature dependence of CTE*_x_*, CTE*_y_*.

Through identical boundary conditions that appear when the equivalent Young’s modulus is extracted, the central point of the FEA model of a microbump unit is fixed to prevent rigid body motion. Moreover, the *x*-directional and *y*-directional nodal displacements of nodes at the corresponding horizontal, vertical, and horizontal edges are joined, respectively. With a uniform incremental Δ*T* in temperature (Figure 5a), the induced thermal strain along the *x*-direction can be obtained from the deformation along the *x*-direction divided by the microbump unit dimension *W*. Therefore, the CTE*_x_* along the *x*-direction can be calculated from the thermal strain divided by the increment in temperature. The gains of equivalent CTE*_y_* along the *y*-direction are implemented by performing the same procedures (Figure 5b).

## 3. Numerical Convergence of Finite Element Model with Equivalent Microbumps

**Figure 6:**
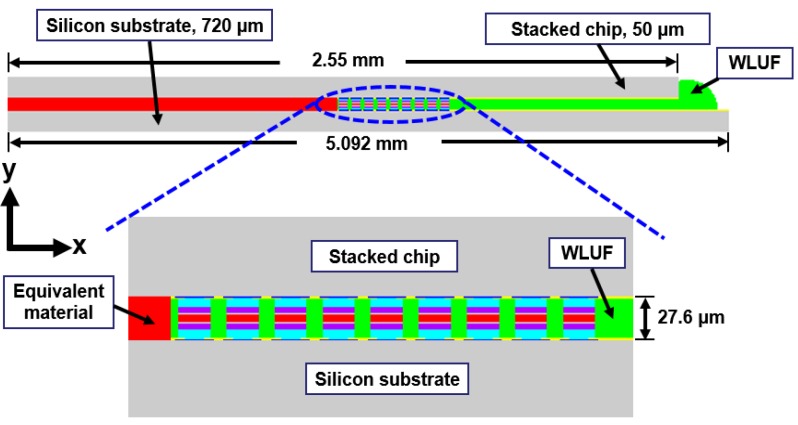
Finite element model of 3D chip stacking with WLUF when equivalent materials of the microbump unit are introduced into the internal part of the packaging structure.

**Figure 7:**
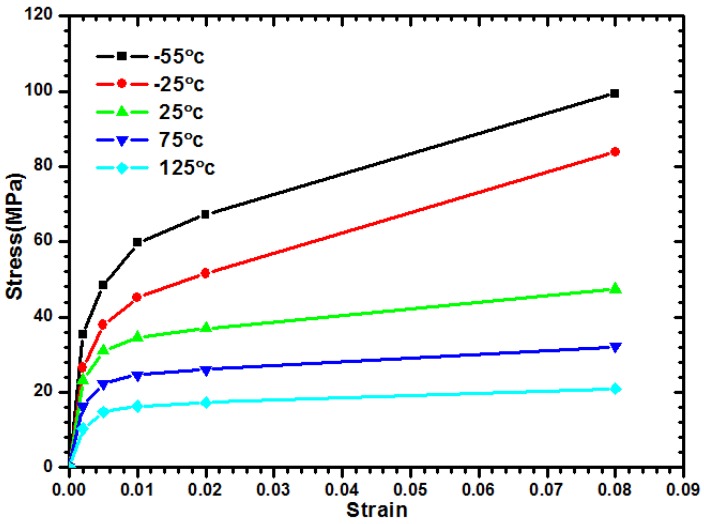
Temperature-dependent stress-strain curve of lead-free solder.

**Figure 8:**
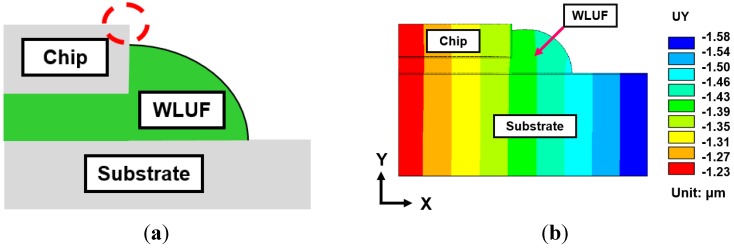
Concerned location of the proposed package with chip stackings: (**a**) outermost top edge of stacked chips; (**b**) enlarged view of the warpage contour of the outermost region of the package with WLUF.

Detailed components of the outermost microbump array need to be constructed in FEA to estimate the real stress and warpage contours on the peripheral edges of a stacked chip resulted from the CTE mismatch between WLUF and microbumps. The converged results influenced by the numbers of outermost actual microbumps needed in a proposed model are investigated to assess the deviated magnitude of estimated plastic strain at the outermost microbump in the packaging model (Figure 6). Under the loading of the temperature cycling test, the model uses materials that are equivalent to those used with a fully constructed chip stacking structure. In the proposed testing vehicle, the cross-section of a packaging assembly with chip stacking cut along the long side of the microbump array is utilized in a 2D FEA. This method demonstrates the effectiveness of the approach of an equivalent material adopted in multi-scale and multi-physical simulation. A 30 μm pitch along this cross-section separates a total of 67 microbumps. The assembled Si chip, with an area of 2.55 mm × 2.55 mm and a thickness of 50 μm, is bonded on a 720 μm thick Si substrate through the WLUF process of thermal compression. The average bump height after assembly is about 27.6 μm. Several assumptions and implements are described in detail from the viewpoint of FEA simulation. Only half-symmetry of the 2D model needs to be constructed because of the bi-axial symmetry of the structure and arrangement in the proposed packaging. Aside from the critical region of the microbump unit, which is far from the packaging center, all the microbump arrays with WLUF are considered a single component with equivalent orthotropic mechanical properties, which were extracted and explained in previous sections. Detailed components of the microbump units at the outermost corner need to be constructed and the corresponding material properties need to be set in FEA. Therefore, the finite element model, which uses the proposed approach of equivalent microbump units, can be presented, as shown in Figure 6. The figure shows nine actual microbump envelopes by WLUF located at the outer region of the microbump array layout. The *x*-directional displacements of nodes at the symmetry axis are fixed to satisfy half-symmetry conditions. All degrees of a node at the bottom of the symmetry axis are constrained to prevent rigid body motion. All material properties used in the finite element model are listed in Table 4. Figure 7 shows the nonlinear material of lead-free solder with the temperature-dependent stress-strain curve. A bi-linear material model is supposed to be copper; others are considered linear elastic. Under a temperature cycling load that ranges between −55 and 125 °C, the warp at the outmost edge of the top surface of the stacked chip is estimated by using the simplified model with an equivalent material of microbump units, as shown in Figure 8. This step is performed to compare the findings with the FEA results, which were obtained with a real microbump array.

**Table 4:** Material properties used in 2D nonlinear FEA.

Materials	Young’s Modulus (*E*)	CTE (ppm/°C)	Poisson’s Ratio
Si	169.5 GPa	3	0.28
Cu	*E* = 122 GPa Yield stress = 173 MPa Tensile strength = 1.2 GPa	17	0.35
Wafer-level Underfill	5.6 GPa	53	0.33
Lead-free solder	Temperature dependence	22.5	0.4
IMC (Ni_3_Sn_4_)	85.6 GPa	17.6	0.31
Ni	186 GPa	12.5	0.342
Al	72 GPa	24	0.36

**Figure 9:**
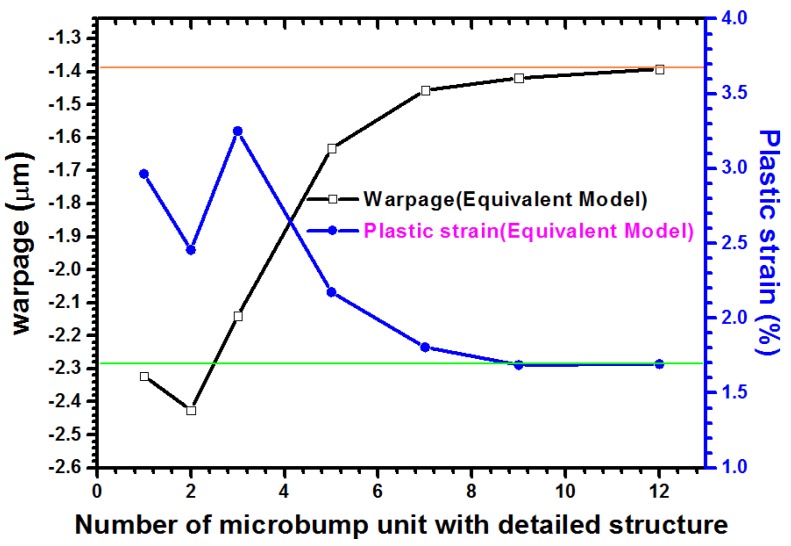
Convergent curves for the maximum warpage of assembled structure and the induced plastic strain of the outermost microbump when real microbump units increased close to the concerned assembled region far from the packaging center.

For an assembled chip with a thickness equal to 50 μm, a ~−1.4 μm warp, as indicated by the displacement contour in Figure 8b, is induced at the chip edge of the packaging structure. When taken as the reference, the converged effect for numerical results *versus* an increase of real microbumps from the outermost location of the bump layout is investigated. The analytic results are shown in Figure 9. When the number of microbump units is larger than eight, the concerned location of warpage gradually reduces from ~−2.3 μm to ~−1.4 μm. In other words, a width of 210 μm (at least seven pitches) is needed for the ring-like region of actual microbumps filled by WLUF, which is located outside the interconnected layout. A similar trend is observed on the lead-free solder region of the outermost microbump with the physical item of plastic strain. The simulated result indicates that plastic strain dropped from 2.96% to 1.69%, and more than 12 real microbump units are achieved. A slight difference is observed between this microbump number and a microbump number equal to eight. Therefore, numerical accuracy of the concerned microbump and package warpage will be achieved when at least eight real microbumps in FEA with equivalent material of microbump unit are arranged at the critical regions of the packaging structure.

## 4. Sensitivity Analysis of Designed Factors in Packaging Structures with WLUF

**Figure 10:**
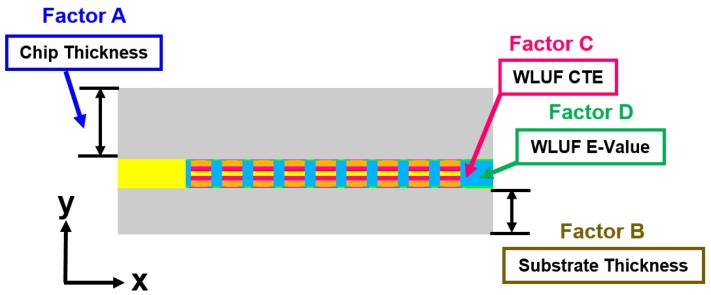
Schematic of locations for four considered factors in the proposed chip stacking structure.

Based on the validated FEA of the packaging model combined with equivalent materials of microbump extracted through previous analysis four factors, namely, chip thickness, substrate thickness, CTE, and E-value of WLUF, are used for three-level factorial designs and subsequent ANOVA. Chip and substrate thicknesses are selected because of the requirements of scaling form factor of a complete packaging structure. Another finding is that the material properties of WLUF clearly affect the warpage and stress-strain distribution of packages during the bonding process and thermo-mechanical reliability tests. Subsequently, the WLUF material is considered in this study. The effect of the single-design factor and the interaction effect between the factors can be statistically qualified through the aforementioned factorial design and ANOVA. A total of 81 runs need to be implemented in the simulation-based analysis. The locations of designed factors in the packaging structure and the magnitude of examined levels are shown in Figure 10 and Table 5. The following sections interpret the detailed analytic results.

**Table 5:** Three levels of factorial designs for concerned factors of packaging structure with WLUF.

Designed Factor	Low Level	Middle Level	High Level
Chip thickness (A)	50 μm	385 μm	720 μm
Substrate (B)	50 μm	385 μm	720 μm
CTE of WLUF (C)	10 ppm/°C	35 ppm/°C	60 ppm/°C
E-value of WULF (D)	1 GPa	7 GPa	13 GPa

### 4.1. Significant Influences of Designed Factors for Warpage

**Figure 11:**
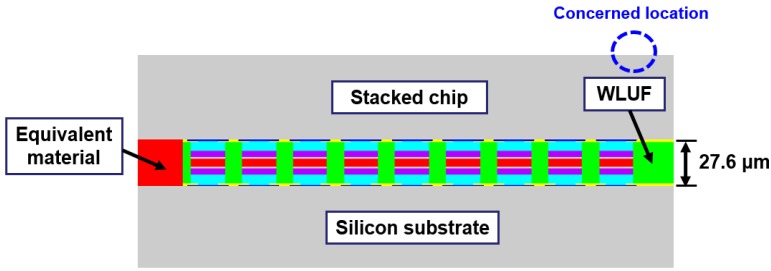
Inspected location of structural warpage considered in factorial analysis.

**Figure 12:**
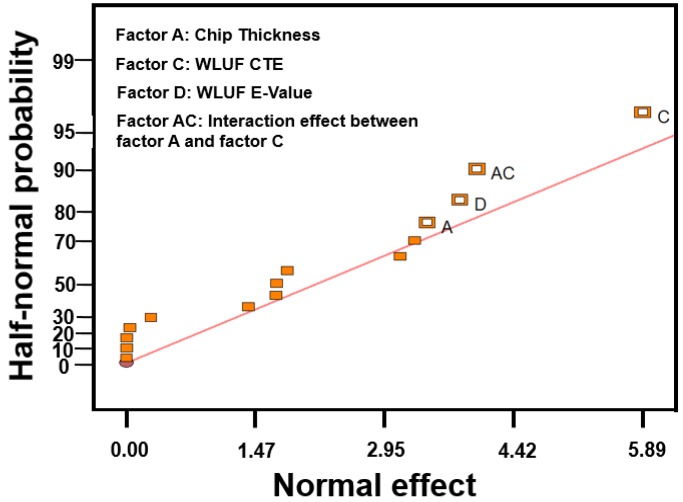
Half-normal probability plots for warpage effect.

**Figure 13:**
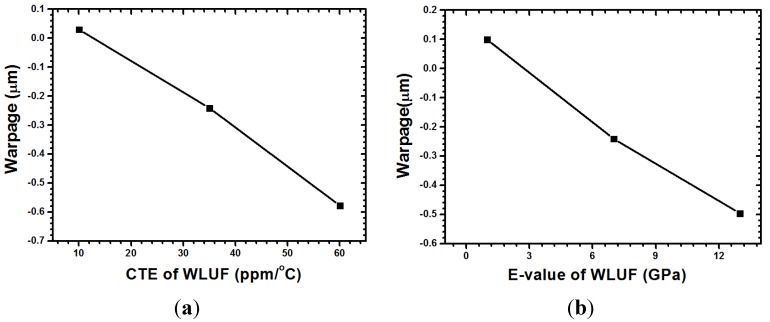
Plots of main effects with other factors fixed at middle level: (**a**) CTE of WLUF; (**b**) *E*-value of WLUF.

The designed factors with regard to the warpage at the concerned location are labeled in Figure 11. According to the half-normal probability plot shown in Figure 12, the most significant factor is CTE of WLUF, followed by the interaction effect between chip thickness and CTE of WLUF. The following item is the E-value of WLUF. The analytic results indicate that the interaction effect has a serious effect on warpage, that is to say, chip thickness and CTE of WLUF cannot be altered independently. To study the main effect of a single factor on warpage, such as CTE of WLUF and E-value, the effects of these factors from the low to the high level is examined, and other designed factors are fixed at the middle level. The plots for the main effects of CTE and E-value of WLUF are shown in Figure 13a,b, respectively. Figure 13a shows that as CTE of WLUF increases from 10 ppm/°C to 60 ppm/°C, the induced warpage rises from 0.03 μm to −0.577 μm. Such behavior is assumed to be due to a significant CTE mismatch between the underfill and stacked silicon chip. When the E-value of WLUF changed from 1 GPa to 13 GPa, warpage is reduced to −0.496 μm. This phenomenon can be attributed to the fact that a softer WLUF easily induces deformation through the flexibility of the chip itself. Consequently, when the E-value of WLUF increases to 13 GPa, a stiff WLUF with a CTE equal to 35 ppm/°C can restrain structural distortion.

**Figure 14:**
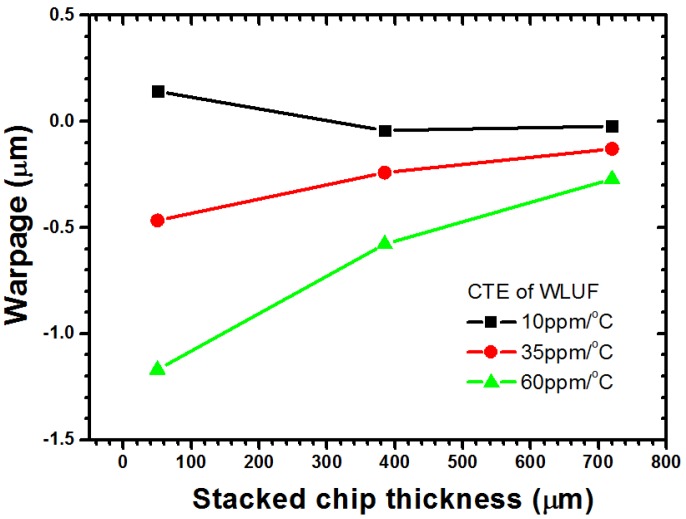
Interaction effect between stacked chip thickness and CTE of WLUF on packaging warpage while substrate thickness and E-value of WLUF are fixed at 50 μm and 7 GPa, respectively.

**Figure 15:**
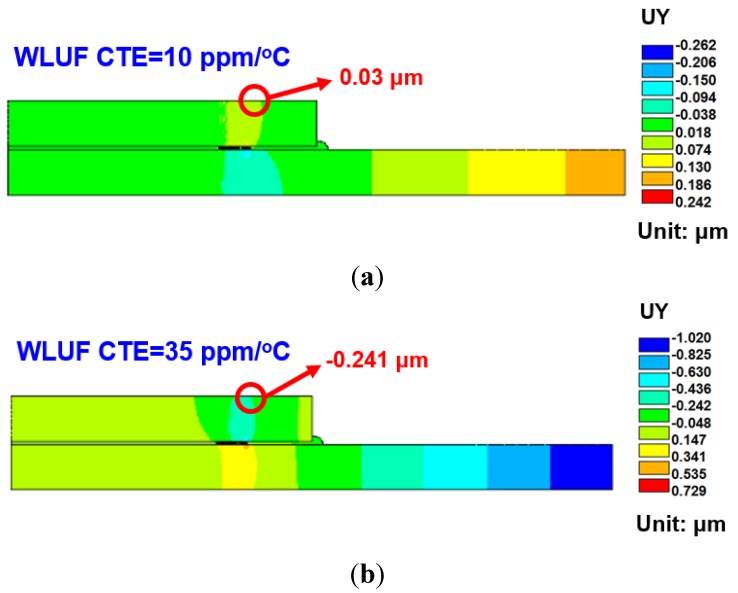
Warpage contour for a deformed/undeformed packaging vehicle with different CTE of WLUF when stacked chip and silicon substrate have same thickness of 385 μm: (**a**) CTE = 10 ppm/°C; (**b**) CTE = 35 ppm/°C.

The interaction effect between stacked chip thickness and CTE of WLUF is plotted in Figure 14. When CTE of WLUF is maintained at 60 ppm/°C, the induced warpage can be reduced from −1.16 μm to −0.27 μm because a stacked chip becomes stiff as its thickness increases from 50 μm to 720 μm. For the other two CTEs of WLUF, similar curves are obtained by the same mechanism. A small warp is acquired when a low CTE of WLUF is applied. Warpage contours for CTEs equal to 10 and 35 ppm/°C are separately plotted in Figure 15.

### 4.2. Equivalent Plastic Strain of Lead-Free Solder Induced in Microbump Interconnect

**Figure 16:**
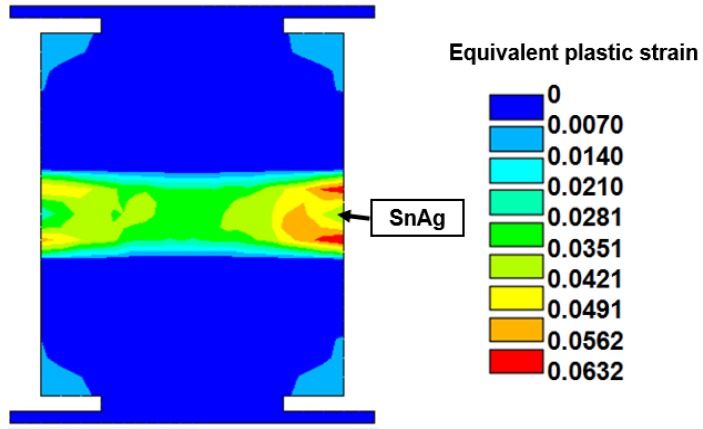
Contour of the equivalent plastic strain after three thermal cycles for the critical microjoint as the thicknesses of stacked chip and substrate are fixed at 385 μm and *E*-value and CTE of WLUF at 7 GPa and 35 ppm/°C, respectively.

**Figure 17:**
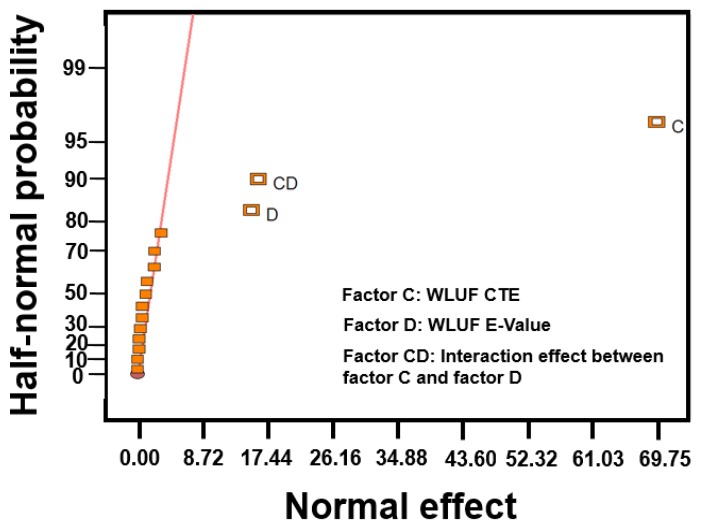
Half-normal probability plot for the equivalent plastic strain of outermost microbump at the proposed packaging structure.

Estimating equivalent plastic strain with consideration of the four designed factors is necessary because solder pastes of microbumps usually dominate the fatigue life of a critical interconnecting joint. Thus, the condition of these solder pastes is the key to fracture failure of an entire package. The maximum equivalent plastic strain at the outermost location of the lead-free micro joint is inspected under a temperature cycling load between −55 and 125 °C. An example of strain contours of lead-free solder is shown in Figure 16. ANOVA and the half-normal probability plot (Figure 17) identified CTE of WLUF as the most significant factor, followed by the interaction effect between CTE and the E-value of WLUF material characteristics. In other words, the mechanical properties of WLUF are the key to improving fatigue micro joint reliability in the packaging structure.

**Figure 18:**
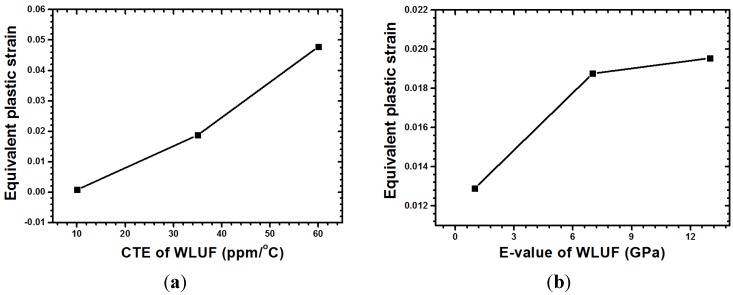
Response of equivalent plastic strain for the main designed factor: (**a**) CTE of WLUF; (**b**) *E*-value of WLUF.

**Figure 19:**
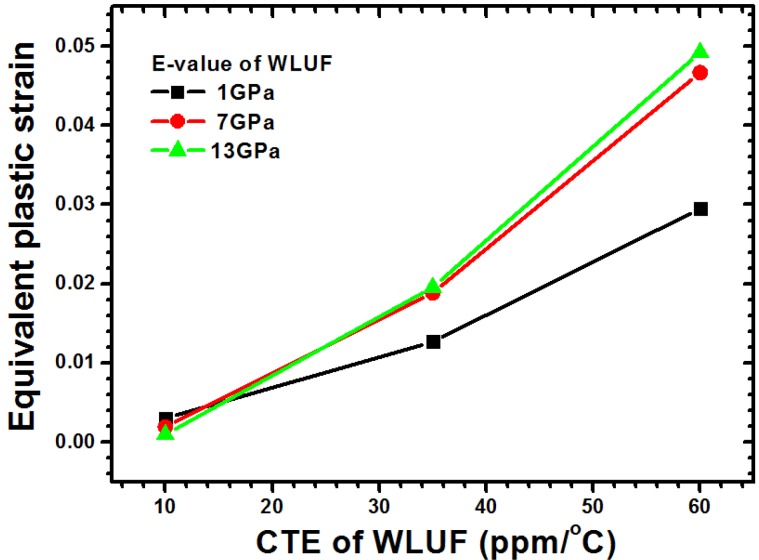
Interaction effect between CTE and *E*-value of WLUF on the equivalent plastic strain of lead-free solder equivalent plastic strain at the outermost microbump.

The corresponding magnitude of plastic strain when considering the main effect of CTE of WLUF is shown in Figure 18a. The analytic results indicate that as a 60 ppm/°C is reached, the equivalent plastic strain of outermost lead-free solder microbump due to thermal mismatch increases up to 0.048 when the CTE of WLUF is equal to 60 ppm/°C. A rigid WLUF has the disadvantage of releasing the strain of microbumps through structural deformation. The aforementioned behavior is especially evident in WLUF with an *E*-value of 13 GPa, as shown in Figure 18b. The results of the interaction effect between CTE and *E*-value of WLUF on the strain magnitude of critical micro joint are shown in Figure 19. Under a constant *E*-value of WLUF, all the curves indicate that a high CTE of WLUF would likely introduce a large strain. In addition, when the CTE of WLUF is equal to 60 ppm/°C, a soft WLUF, which is regarded as a buffer layer, can help to release microbump strain. However, a hard WLUF with a low CTE, such as 10 ppm/°C, is used to protect microbumps. This study recommends the use of rigid WLUF that has a low CTE in the chip stacking structure.

## 5. Conclusions

This paper proposes the use of an equivalent material to replace a real, complicated microbump unit in an assembly structure. This approach overcomes the difficulty of modeling construction, which arises because of a significant scale mismatch among 3D-IC packages. Moreover, this approach enhances the converged capability and accuracy in numerical aspects. Non-linear stress-strain curves and related mechanical properties for equivalent material, such as equivalent Young’s modulus, Poisson’s ratio, shear modulus, and CTE, are obtained by performing FEA under given loads of corresponding traditional measured tests of bulk materials. The proposed approach is beneficial in acquiring and maintaining the mechanical characteristics of temperature dependence. The workability and reliability of the approach of extracting equivalent material for a microbump unit need to be proven. Thus, an estimated comparison is performed between package warpage and the plastic strain of critical bump between the detailed components of packaging vehicle and simplified simulated model of the same structure, including equivalent material of the microbump unit, respectively, under temperature cycling loads. Results indicate that if the converged results for the case with equivalent microbump units are satisfied, then a ring-like region composed of at least eight real microbumps is needed at the outermost location of a stacked chip. The factorial design indicates that the most significant factor is the CTE of WLUF, followed by the E-value of WLUF. In addition, the interaction effect between stacked chip thickness and CTE of WLUF must be considered. Low CTE of WLUF is suggested when enhancing the assembly and thermo-mechanical reliability of the present packaging structural design. This paper provides a promising and valuable computational mechanics solution to deal with the complexity and polytropy of future 3D-IC packages.
